# Cavity-Modified Chemiluminescent Reaction of Dioxetane

**DOI:** 10.1021/acs.jpca.3c05664

**Published:** 2023-10-17

**Authors:** Mahesh Gudem, Markus Kowalewski

**Affiliations:** Department of Physics, Stockholm University, Albanova University Centre, SE-106 91 Stockholm, Sweden

## Abstract

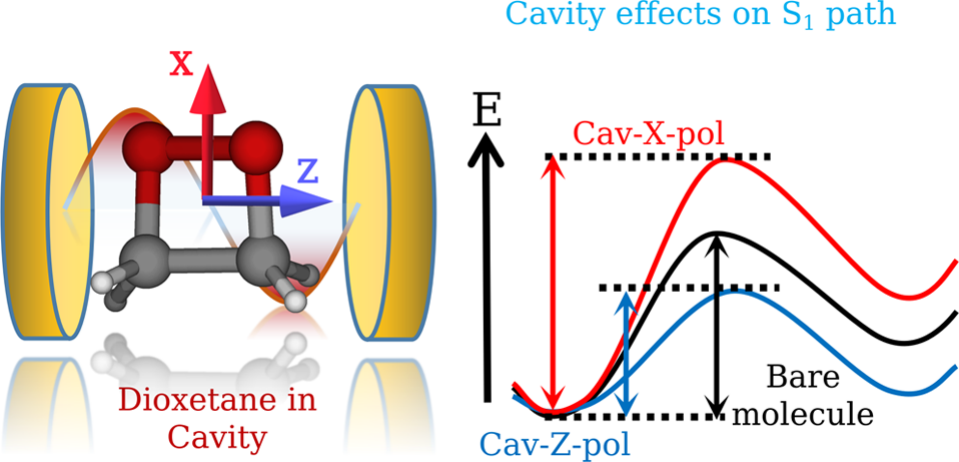

Chemiluminescence is a thermally activated chemical process that emits a photon of light by forming a fraction of products in the electronic excited state. A well-known example of this spectacular phenomenon is the emission of light in the firefly beetle, where the formation of a four-membered cyclic peroxide compound and subsequent dissociation produce a light-emitting product. The smallest cyclic peroxide, dioxetane, also exhibits chemiluminescence but with a low quantum yield as compared to that of firefly dioxetane. Employing the strong light–matter coupling has recently been found to be an alternative strategy to modify the chemical reactivity. In the presence of an optical cavity, the molecular degrees of freedom greatly mix with the cavity mode to form hybrid cavity–matter states called polaritons. These newly generated hybrid light–matter states manipulate the potential energy surfaces and significantly change the reaction dynamics. Here, we theoretically investigate the effects of a strong light–matter interaction on the chemiluminescent reaction of dioxetane using the extended Jaynes–Cummings model. The cavity couplings corresponding to the electronic and vibrational degrees of freedom have been included in the interaction Hamiltonian. We explore how the cavity alters the ground- and excited-state path energy barriers and reaction rates. Our results demonstrate that the formation of excited-state products in the dioxetane decomposition process can be either accelerated or suppressed, depending on the molecular orientation with respect to the cavity polarization.

## Introduction

**Figure 1:**
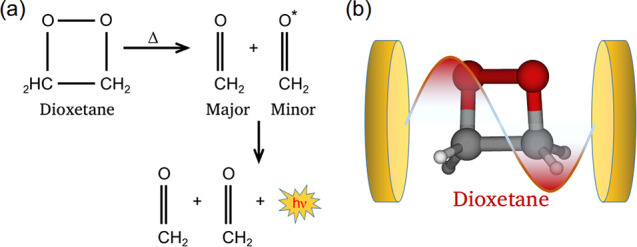
(a) Thermal decomposition of dioxetane resulting the formation of ground- and excited-state formaldehyde fragments. (b) Schematic display of dioxetane inside an optical cavity.

Chemiluminescence is a fascinating phenomenon in which a thermal reaction generates a light-emitting product,^1^ also termed bioluminescence if the photon-generating chemical reaction occurs in a living organism.^2^ This cold light emission process has been utilized for several biological and chemical applications such as genetic engineering,^3^ bioluminescence imaging,^4^ pyrosequencing,^5^ nitric oxide analyzer,^6−8^ and forensic serology to identify blood traces.^9,10^ In nature, there are a number of bioluminescent systems, mainly marine organisms like jellyfish, crustaceans, sea stars, and algae, but also terrestrial creatures such as the famous firefly beetle and some worms.^11−15^ The bioluminescence phenomenon mainly involves the enzymatic conversion of luciferin substrate into a high-energy peroxy intermediate, called 1,2-dioxetane (termed dioxetane hereafter).^16,17^ This four-membered cyclic compound has been observed to be a common chemical moiety that exists in most chemiluminescent and bioluminescent reactions. Interestingly, dioxetane itself exhibits chemiluminescence, however, with a relatively smaller quantum yield compared to the bioluminescence process in firefly. Being the simplest and smallest chemiluminescent system, the thermal decomposition of dioxetane has been extensively investigated by several experimentalists and theoreticians.^18−30^ Upon thermal activation, the molecule dissociates into two formaldehyde fragments, among which a small fraction will be produced in the excited electronic state and the subsequent radiative decay to the electronic ground state emits a photon (Figure 1a). The experimental and theoretical studies have shown that most of the excited state products of the dioxetane dissociation end up in a nonemissive triplet state, which results in the low chemiexcitation efficiency.^18,28,30,31^ Numerous dioxetane derivatives were explored with the intention to increase the quantum yield of chemiluminescence.^32,33^ Alkyl and aryl groups, without electron-donating substituents, were found to increase the triplet chemiexcitation yield.^26^ The electron-donating functional group, i.e., phenolate (deprotonated phenol anion) ion, in turn, causes the dioxetane molecule to decompose quickly and efficiently to form singlet excited state products, resulting in intense light emission.^34,35^ The fast dissociation here might create additional difficulties in synthesizing these dioxetane derivatives.

In recent years, strong light–matter coupling in optical nanocavities has been found to be the alternative tool for controlling the chemical reactions.^36−50^ The strong coupling of the cavity vacuum field with the molecular electronic or vibrational degrees of freedom generates hybrid light–matter states called polaritons. These dressed states can significantly alter the potential energy surfaces (PESs) and modify the chemical reactivity of the considered system, such as enhancing, suppressing, or even introducing new reaction pathways.^43,45,46,51−55^ Several experimental and theoretical studies have demonstrated the potential applications of strong light–matter coupling in thermal and photochemical processes. For instance, polaritonic states are exploited in manipulating intramolecular vibrational energy transfer processes,^56−58^ reaction rates,^55,59,60^ branching ratio between different reaction paths,^45,61^ and exciton harvesting and exciton transport,^62,63^ just to mention a few examples. Along with outstanding applications, a wide range of theoretical methods have also been developed to understand the underlying principles of polaritonic chemistry.^37,38,41,49,64−71^ They include quantum optics,^49,55,72−74^ semiclassical approaches,^75^ orbital theories,^76^ and *ab initio* quantum electrodynamics (QED) methods.^77−82^

In this work, our aim is to understand the modifications in the energetics of the dioxetane chemiluminescent reaction in the presence of an optical cavity (Figure 1b). During the thermal decomposition process of the bare molecule, multiple electronic states become degenerate, resulting in a population of excited electronic states. Therefore, the cavity-modified ground- and excited-state minimum energy paths (MEPs) for dioxetane dissociation have been computed by employing the cavity-coupled molecular Hamiltonian. We explore how strong light–matter coupling changes the activation energies and the corresponding reaction rates. Here, we use extended molecular Jaynes–Cummings model to calculate the cavity PESs corresponding to the different electronic states.^38,51,52^ In this model, the electronic properties of the molecule are calculated without explicitly including the light–matter interaction but rather are considered as a correction term to the bare molecular Hamiltonian. The resulting PESs obtained diagonalizing the cavity-coupled molecular Hamiltonian parametrically depend on both molecular and photonic coordinates. This approach has been successfully applied in molecular systems to understand the influences of the cavity on the chemical reactivity.^38,51,52^ Alternatively, QEDFT^83^ and QED-CC^84−86^ methods, where the cavity–matter interaction is explicitly considered in the electronic structure calculations, have also been used to study the effect of cavity on ground state chemical processes.^87,88^

The present study considers the interaction of a single cavity mode with a single molecule, i.e., collective coupling effects that exist in the case of experimental polaritonic chemistry studies are not included here. It should be noted that recent experiments have demonstrated that strong field–matter interaction can in principle be achieved under the single molecule coupled to a cavity mode condition.^89−94^ In this paper, we investigate theoretically which coupling parameters are required to achieve a sizable effect, even if they may lie outside the currently achievable experimental parameter regime. We also mention here that the cavity effects have been explored for different orientations of the molecule with respect to the cavity mode polarization. Along with the collective coupling effects, cavity losses and dephasing effects should eventually be considered in describing the cavity-induced modifications on the chemiluminescent reaction of dioxetane.

## Theory and Methods

### Model Hamiltonian

The general form of light–matter coupling is covered by the multipolar formulation of QED.^95−98^ Here, we use a nonrelativistic Pauli–Fierz Hamiltonian in the length gauge^99^ to describe the interaction of matter with the cavity mode. The Hamiltonian (*Ĥ*_MC_) for the considered system, a molecule coupled to a single cavity mode, in length gauge and dipole approximation is given by the sub-Hamiltonians of molecule (*Ĥ*_M_), cavity (*Ĥ*_C_), and the light–matter interaction term (*Ĥ*_I_). All of the equations are presented in atomic units if not stated otherwise.
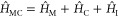
1The molecular Hamiltonian *Ĥ*_M_ consists of the potential (*V̂*) and nuclear kinetic energy (*T̂*_N_) operators. The cavity mode Hamiltonian *Ĥ*_C_ with cavity frequency ω_c_, generally treated as a harmonic oscillator, in the photon displacement coordinate representation reads as
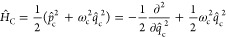
2Here, *q̂*_c_ and *p̂*_c_ are the photon displacement coordinate and conjugate momentum, respectively. By using the bosonic photon creation (*â*_c_^†^) and annihilation (*â*_c_) operators, *q̂*_c_ and *p̂*_c_ can be written in second quantization formalism as
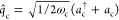
3
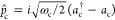
4The light–matter interaction term *H*_I_ in the photonic coordinate representation is given as^38,51,52^
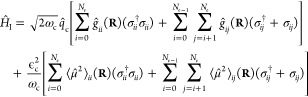
5Here, first two terms describe the light photon–matter coupling, where *g*_*ii*_ and *g*_*ij*_ correspond to the coupling strengths of the cavity mode with the molecular vibrational and electronic degrees of freedom, respectively. The last two terms represent the dipole self-energy (DSE) interaction, which appears due to the length-gauge transformation. The DSE interaction term can be interpreted as the effect of molecular polarization on the cavity photon field. As can be seen in eq 5, the DSE interaction term of the Pauli–Fierz Hamiltonian depends on the magnitude of the cavity radiation field ε_c_. However, it is often ignored while studying the cavity-modified chemistry by using the Pauli–Fierz Hamiltonian, but many of the recent studies have demonstrated the strong influence of this interaction on the ground and excited state chemical properties.^51,99,100^

The photon displacement coordinate description of the cavity Hamiltonian employed here provides a convenient way to represent the cavity mode.^38,51,52,101^ In this representation, the cavity-coupled molecular potential energy surfaces depend parametrically on nuclear and photonic coordinates. Furthermore, the interaction Hamiltonian does not invoke the rotating-wave approximation that neglects the counter rotating terms (*â*_c_σ_*ij*_ and *â*_c_^†^σ_*ij*_^†^), which is in contrast to the widely employed Jaynes–Cummings model.^72^ The strength of electronic and vibrational cavity couplings depends on the corresponding Rabi frequency
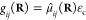
6Here,  denotes the field strength of the optical cavity with volume *V*_c_, and μ̂_*ij*_(**R**) are the permanent (for *i* = *j*) and transition (for *i* ≠ *j*) dipole moments along the molecular reaction coordinate **R**. As can be seen from the definition, the magnitude of ε_c_ depends on the effective volume *V*_c_ of the cavity and, thus, the confinement of the cavity mode. In eq 5, σ_*ij*_ = |*i*⟩⟨*j*| (σ_*ij*_^†^ = |*j*⟩⟨*i*|) represents the annihilation (creation) operator of the electronic excitation, and ⟨μ̂^2^⟩ is the expectation value of the squared dipole operator. In total, eight electronic states (four singlets and four triplets), i.e., *N*_*e*_ = 8, were considered in the present study. Note that the singlet-to-triplet conversion is spin-forbidden, resulting in a zero transition dipole moment. Because of this property, the electronic cavity coupling between singlet and triplet states in the interaction term *Ĥ*_I_ of our model Hamiltonian is considered to be zero. The four singlets (S_0_–S_4_) and the four triplets (T_1_–T_4_) are independently coupled to the cavity field mode, resulting in a block-diagonal cavity–molecule Hamiltonian.

The current study varies the parameters ε_c_ and ω_c_ to understand the cavity influences on the chemiluminescence process of dioxetane. Furthermore, the cavity effects were investigated for two different molecular orientations with respect to the cavity field polarization (see below for more details), and a single molecule has been coupled to the cavity mode. Note that the polaritonic experiments usually consider cavities with many molecules, rather than a single one, to attain the strong coupling regime. In this scenario, the collective cavity coupling strength increases by √*N*, with *N* being the number of molecules, and the corresponding increase in the Rabi splitting can be observed in the transmission spectra. It should be mentioned that the collective coupling effects are not simply rescaling of the cavity coupling but also other effects due to the dark states formed by the coupling of many molecules to the cavity mode. Investigating the impact of such dark states on the chemiluminescence process is an interesting area for future research. In addition, structural modifications of the molecule in the presence of an optical cavity should also be included in further extension of the study.

### Computational Methods

All stationary points on the ground and excited electronic states were obtained using the state-specific (SS) CASPT2^102,103^ method along with the def2-TZVPP basis set. The corresponding reference wave function includes four states in the state-averaged CASSCF^104−106^ for the singlet (S_0_–S_3_) and triplet (T_1_–T_4_) manifolds separately. The active space used consists of 12 electrons distributed among 10 orbitals (Figure S1 in the Supporting Information) in which the bonding and antibonding orbitals of C–C′, O–O′, C–O, and C′–O′ bonds along with the two lone-pair orbitals on the oxygen are considered as the active orbitals. The same state-averaged CASSCF was also used in previous studies, where equal weights of four roots with a 12-in-10 active space were shown to be essential to adequately describe the complete dissociation profile of dioxetane.^28,30^ The potential energies along the linearly interpolated internal coordinate (LIIC) paths connecting the optimized structures were computed using the MS-CASPT2^107,108^ method along with the def2-TZVPP basis set. In the reference CASSCF of the MS-CASPT2 calculation, eight states (S_0_–S_3_ and T_1_–T_4_) with equal weights were included in the state-averaged wave function. For both the SS-CASPT2 and MS-CASPT2 calculations, the core orbitals of non-hydrogen atoms were considered frozen, and an IPEA shift of 0.25 was employed to avoid the intruder state problem. All the electronic structure calculations were performed by using the MOLPRO-2021 program package.^109,110^

The expectation value of the squared dipole operator ⟨μ̂^2^⟩_ij_ used to compute the DSE has been obtained by using the dipole moments in the following resolution of identity equation:^51,111^
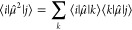
7where μ̂ represents the dipole operator and *k* runs over all the considered electronic states. Note that the expectation value of the squared dipole operator has been computed for singlet and triplet states separately, i.e., the dipole moments corresponding to four singlets (S_0_–S_4_) and four triplets (T_1_–T_4_) have been used for the case of singlet and triplet manifolds, respectively

To fulfill the zero-field condition,^112−114^ the energy is minimized along *q̂*_c_ for each value of **R**. These minimum energy points were used to represent the cavity-modified MEP between the reactant and product. The MEP was located on both ground and excited state PESs and was subsequently used to estimate the energy barriers and reaction rates.

The influences of the cavity on the reaction rate were computed using the Arrhenius equation, which depends on the energy barrier between the reactant and product. Ideally, dynamics simulations need to be performed for an accurate theoretical estimation of the reaction rates, as reported in previous theoretical studies.^38,51,52,61,115^ However, they may become computationally expensive for the considered dissociation reaction, primarily because of the several electronic states involved (both singlet and triplet) along the chemiluminescence process and corresponding computation of gradients, nonadiabatic couplings, and spin–orbit couplings. Furthermore, the computed ground and excited state potential energy curves are not completely repulsive, but they exhibit several minima, as will be discussed below. Consequently, the nuclear motion needs to be evolved for much longer time scales, which would also increase the computational cost. We thus focus here on the analysis of stationary points and reaction pathways. It should also be noted that the Arrhenius equation describes the rate of a thermal reaction involving an ensemble of molecules. The present study, however, considers the scenario of a single molecule coupled to a cavity mode. The reaction rates computed here are intended to provide a qualitative understanding of the cavity influences on the reaction rates due to the modified energetics at room temperature. This rate equation was utilized for the same purpose in previous studies.^116,117^ In the present work, reaction rates for the excited state pathways were also computed using the Arrhenius rate equation. This is because the considered chemical process is a thermally activated chemiluminescent reaction that involves multiple electronic states, as discussed above, even in the absence of an external light field. The change in the reaction rate due to the presence of an optical cavity can be estimated by the ratio of the rate constants for the molecular system with (*k̃*) and without (*k*) the strong light–matter interaction:
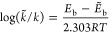
8Here, *E*_b_ and *Ẽ*_b_ represent the reaction energy barriers (in joules) for the bare molecule and the hybrid cavity–matter system, respectively. *R* is the ideal gas constant, and *T* denotes the temperature, which is assumed to be 300 K.

## Results and Discussion

Before discussing the influences of the cavity on the dioxetane chemiluminescence reaction, we first provide details of the calculated energy profiles for the dissociation of the bare molecule. The chemiluminescence mechanism in dioxetane is well-established based on experimental and high-level theoretical studies. Therefore, we briefly discuss the mechanism and compare the energetics and geometrical parameters of the optimized structures with previously reported data.

In earlier studies by Lindh and co-workers, several stationary points were optimized in the ground and excited electronic states (S_1_ and T_1_) for the thermally activated decomposition of dioxetane.^28,30^ Here, we employed the SS-CASPT2/TZVPP method to obtain the relevant geometries on the PESs. They include reactant minimum (S_0_-react), ground state saddle point (S_0_-TS), minima on S_1_ and T_1_ states (S_1_-min and T_1_-min), singlet and triplet transition states (S_1_-TS and T_1_-TS), and dissociated products on S_0_, S_1_, and T_1_ states (S_0_-prod, S_1_-prod, and T_1_-prod). The important geometrical parameters, C–C′ and O–O′ bond lengths, and the O–C–C′–O′ dihedral angle of these stationary points are collected in Table S1 in the Supporting Information. The coordinates of the reported structures from MS-CASPT2 optimizations^28^ are also provided for comparison. As can be seen in the table, the optimized geometries of the two methods have been found to be very similar with minor structural differences. On the S_1_ (and T_1_) PESs of dioxetane, one more set of minimum and transition state structures were reported in the previous theoretical studies by Lindh and co-workers.^28,30^ These additional stationary points nearly have the same energy as the above-mentioned S_1_-min (T_1_-min) and S_1_-TS (T_1_-TS) but mainly differ in the dihedral angle as ≈180° instead of ≈70°. As will be discussed below, the excited states in the thermal dissociation process of dioxetane are accessible around the S_0_-TS geometry, at which the dihedral angle is ≈43°. From this region of PES, the molecule on the excited state is expected to reach the ≈70° minimum first, rather than the ≈180° structure. The present study therefore considers the excited state pathways involving the structures with the dihedral angle of ≈70°, and the ≈180° geometries have not been optimized. Note that the S_1_-min (T_1_-min) and S_1_-TS (T_1_-TS) in this work are denoted as Min_S1(70)_ (Min_T1(70)_) and TS_S1(70)_ (TS_T1(70)_), respectively, in the theoretical study of Farahani et al.^28^

**Figure 2:**
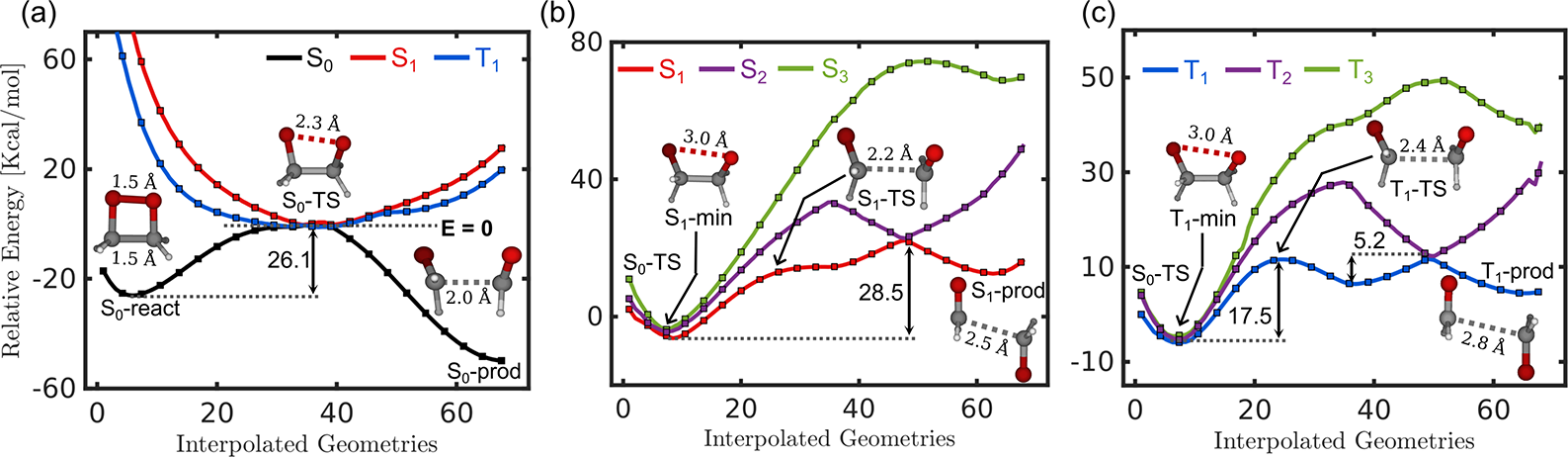
MS-CASPT2/TZVPP computed energy profiles for (a) S_0_ path connecting reactant (dioxetane) and dissociative products (formaldehyde), (b) S_1_ path starting from S_0_-TS toward the formation of the S_1_ state product, and (c) triplet path for the formation of T_1_-prod. Here S_0_-TS has been used as the reference geometry, i.e., energy (E) of the S_0_-TS along the paths in (a), (b), and (c) is considered to be zero. The dashed lines and double-sided arrows are used to indicate the energy barriers for the corresponding paths. Significantly changing bond distances at optimized stationary points are given in angstroms.

The MEPs connecting the aforementioned geometries were also explored. However, several theoretical challenges were involved in the corresponding MEP search computations.^30^ The MS-CASPT2//CASSCF energy profiles show some unphysical features such that a MS-CASPT2//MS-CASPT2 treatment was necessary for calculating the ground state path. With regard to the S_1_ PES, even obtaining the MEP was found to be impossible due to the strong degeneracy with the S_0_ state. To avoid these difficulties, we used the LIIC method to connect the optimized critical points and find the ground- and excited-state pathways that form formaldehyde as the dissociative product. Subsequently, the MS-CASPT2 energies for different electronic states have been calculated along the LIIC-constructed paths, and the corresponding energy profiles are shown in Figures 2a, 2b, and 2c for the S_0_, S_1_, and T_1_ states, respectively. The energy barrier for the ground state path has been calculated to be 26.1 kcal/mol, which is in agreement with the value of 24.1 kcal/mol for the MS-CASPT2//MS-CASPT2 computed MEP.^30^ Similar to the previous reports,^28,30^ multiple electronic states, both singlet and triplet, become energetically close to the S_0_ state during the dissociation process of dioxetane (Figure 2a; see Figure S2 in the Supporting Information for more states along the S_0_ path). At the S_0_-TS, the S_1_ and T_1_ states are almost degenerate with the ground electronic state, and the spin–orbit coupling between T_1_ and S_0_ has also been found to be significant.^28^ Consequently, the lowest singlet and triplet excited states are efficiently populated around the degenerate region of the PES.

After reaching the S_1_ (T_1_) state, the molecule first relaxes to the S_1_-min (T_1_-min) and then proceeds through the S_1_-TS (T_1_-TS) to form excited formaldehyde. The calculated energy difference between the SS-CASPT2 optimized minimum and transition state of the S_1_ (T_1_) PES has been found to be 21.5 (15.1) kcal/mol. These values are slightly higher (about 4 kcal/mol) than those at the MS-CASPT2//MS-CASPT2 level of theory.^28^ Based on the LIIC paths, the S_1_ and T_1_ state barriers, the energy difference between the highest and lowest points on the path, have been found to be 28.5 and 17.5 kcal/mol, respectively. As shown in Figure 2b,c, the excited state paths also exhibit curve crossings in the region close to the products. In the case of S_1_ PES, the S_2_/S_1_ crossing is the highest energy point along the path resulting the corresponding barrier significantly larger than the one calculated from the S_1_-TS. On the other hand, the T_2_/T_1_ intersection has less effect on the T_1_ state barrier. Note that there exists another minimum along the T_1_ path from which the intersection point is accessible with a barrier of 5.2 kcal/mol. This value is relatively lower than the one (17.5 kcal/mol) at the T_1_-TS, and therefore the initial step from T_1_-min to the second minimum is expected to be the rate-determining step for the formation of the T_1_ product. Despite the additional second barrier, the T_1_ dissociation is more favorable than that of S_1_, which is due to the overall barrier for the former being lower than that for the latter. As a result, a major fraction of the excited state products form through the triplet channel. This is consistent with the results of Lindh and co-workers and explains the experimentally observed higher triplet quantum yield in the thermal dissociation of dioxetane.^28,30^

In the following, we discuss the influence of cavity–molecule coupling on the ground and excited state PESs, activation energies, and reaction rates. Here, the excited state paths considered proceed through the structures with a dihedral angle of ≈70°; that is, the alternative channels involving Min_S1(180)_ (Min_T1(180)_) and TS_S1(180)_ (TS_T1(180)_) are not included. Furthermore, the constructed reaction energy profiles will not include the entropic trapping effect, which has been shown to be important in efficiently accessing the S_1_ and T_1_ channels in the dioxetane dissociation process.^30^

**Figure 3:**
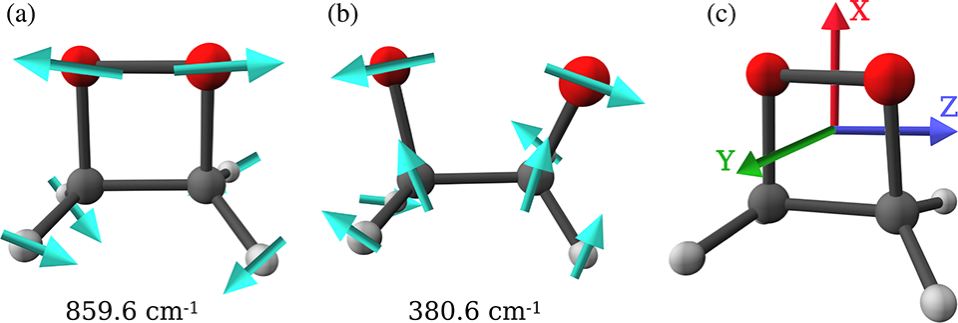
(a) S_0_-react geometry with normal mode corresponding to the reaction coordinate of dioxetane dissociation. (b) S_0_-TS geometry with the imaginary frequency normal mode vector. (c) The molecular coordinate frame used throughout the text. The *X* and *Z* directions are along C–O and C–C/O–O bonds, respectively, and lie in the plane of the cyclic ring, whereas the *Y* direction is out-of-plane to the ring. The values below the structures in (a) and (b) are the normal-mode frequencies in cm^–1^.

The effects of the cavity on the reaction pathways discussed above have been investigated by varying the field strength of the cavity ε_c_ at different frequencies ω_c_. The cavity mode is resonant with the energies ranging from 103.4 to 54.4 meV, which correspond to the normal-mode frequencies of the reaction coordinate at the Franck–Condon point and S_0_-TS geometries, respectively (Figure 3a,b). The electronic and vibrational cavity couplings along with DSE terms have been incorporated into the Hamiltonian used to obtain the cavity PESs as a function of the molecular reaction coordinate and cavity coordinate *q*_c_. Note that our model does not consider resonance effects between molecular vibrational modes and the cavity photon mode. Two different sets of PESs are obtained: one with the cavity light polarized along the *X*-direction and the other along the *Z*-direction, i.e., *X* and *Z* components of dipole moments were used to calculate the corresponding light–matter coupling terms. These cavity field orientations will be termed cav-X-pol and cav-Z-pol, respectively, in the rest of the text. For the quantized light mode polarized along the *Y*-direction, the influence of the cavity has been found to be insignificant and is therefore not discussed further. In the considered coordinate system, the *X*- and *Z*-axes are along C–O and C–C′/O–O′ bonds, respectively, and stay in the plane of the dioxetane ring, while the *Y*-axis goes out-of-plane to the cyclic ring (Figure 3c). The geometrical distortions (O–O′ and C–C′ bond cleavages) involved in accessing the optimized transition state geometries are within the plane of the peroxide ring (Figure 2). Accordingly, the *X* and *Z* components of dipole moments change significantly along the obtained paths, and the out-of-plane *Y* component is nearly unaffected. This also explains the observed small cavity effects when the field polarization is aligned with the *Y*-axis.

**Figure 4:**
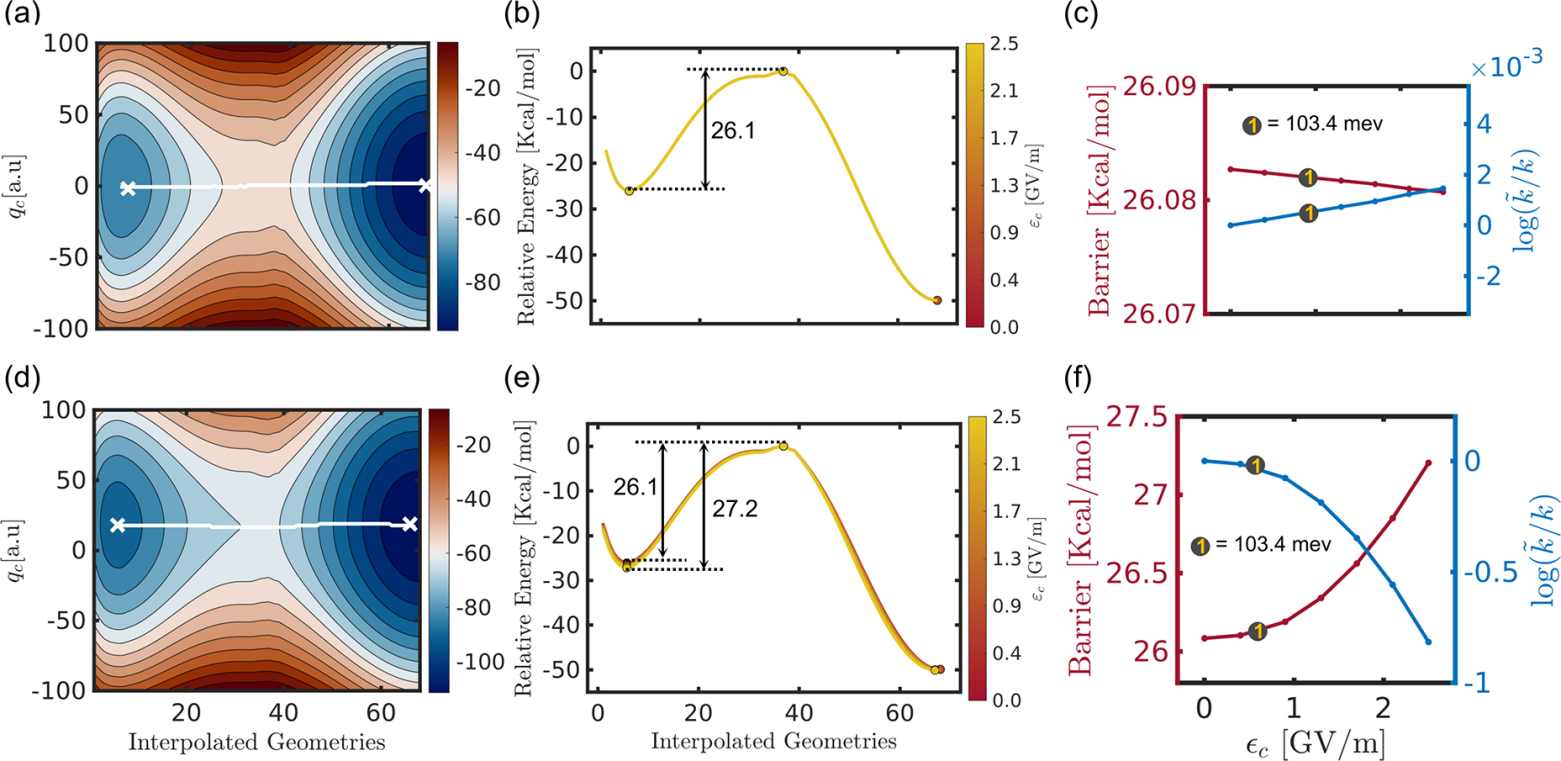
(a) Two-dimensional S_0_ PES in the space of the molecular reaction coordinate and the photonic displacement coordinate of the cavity **q̂**_c_ at ε_c_ = 1.3 GV/m and ω_c_ = 103.4 meV. The white curve represents the MEP along the molecular coordinate. (b) MEP curves at different cavity field strengths ranging from 0 to 2.5 GV/m at ω_c_ = 103.4 meV. (c) Effect of ε_c_ on the energy barrier (red curve) and the rate ratio (blue curve) for the MEPs in (b). The description for (d), (e), and (f) is similar to (a), (b), and (c), respectively, except that the cavity mode is polarized along the *Z* direction (along the C–C′/O–O′ bond) for the former and the *X* direction (along the C–O bond) for the latter.

Figures 4a and 4d depict the ground state PESs at ω_c_ = 103.4 meV and ε_c_ = 1.3 GV/m for cav-Z-pol and cav-X-pol, respectively. The white curve embedded in the PES represents the calculated MEP. It has been observed that the light–matter interaction leads to the manipulation of PESs and results in the shift of MEP along *q*_c_, particularly in the case of cav-X-pol. To evaluate the polaritonic effects on the barrier for the ground state path, the cavity PESs are obtained for a set of cavity field strengths ε_c_ up to 2.5 GV/m at ω_c_ = 103.4 meV. The corresponding MEPs are shown in Figure 4b,e. For cav-Z-pol, the barrier for the ground state path is almost similar to that of the bare molecule. The cav-X-pol lowers the reactant and product minima, while the position of the transition state remains unchanged. This leads to an increase in the barrier from 26.1 kcal/mol for the bare molecule to 27.2 kcal/mol at the highest cavity field strength. We also estimated the rate ratio (log(*k̃*/*k*)) by using the Arrhenius equation (eq 8), which provides the change in the reaction rate with respect to ε_c_. Here, *k̃* and *k* represent the Arrhenius rate constants for the cavity–molecule system and the bare molecule, respectively. As shown in Figure 4c, the negligible change in the barrier with respect to ε_c_ for cav-Z-pol causes the corresponding reaction rates to be nearly the same as those in the bare molecule. The cav-X-pol, in contrast, increases the barrier and reduces the rate by ≈6 times (Figure 4f). With this alignment of the cavity mode, the resonance frequency ω_c_ has also been found to influence the MEPs and the corresponding barriers. The reaction rate gets further decreased by changing ω_c_ from 103.4 to 54.4 meV (Figure S3 in the Supporting Information), while it still remains the same as the bare molecule for the cav-Z-pol.

**Figure 5:**
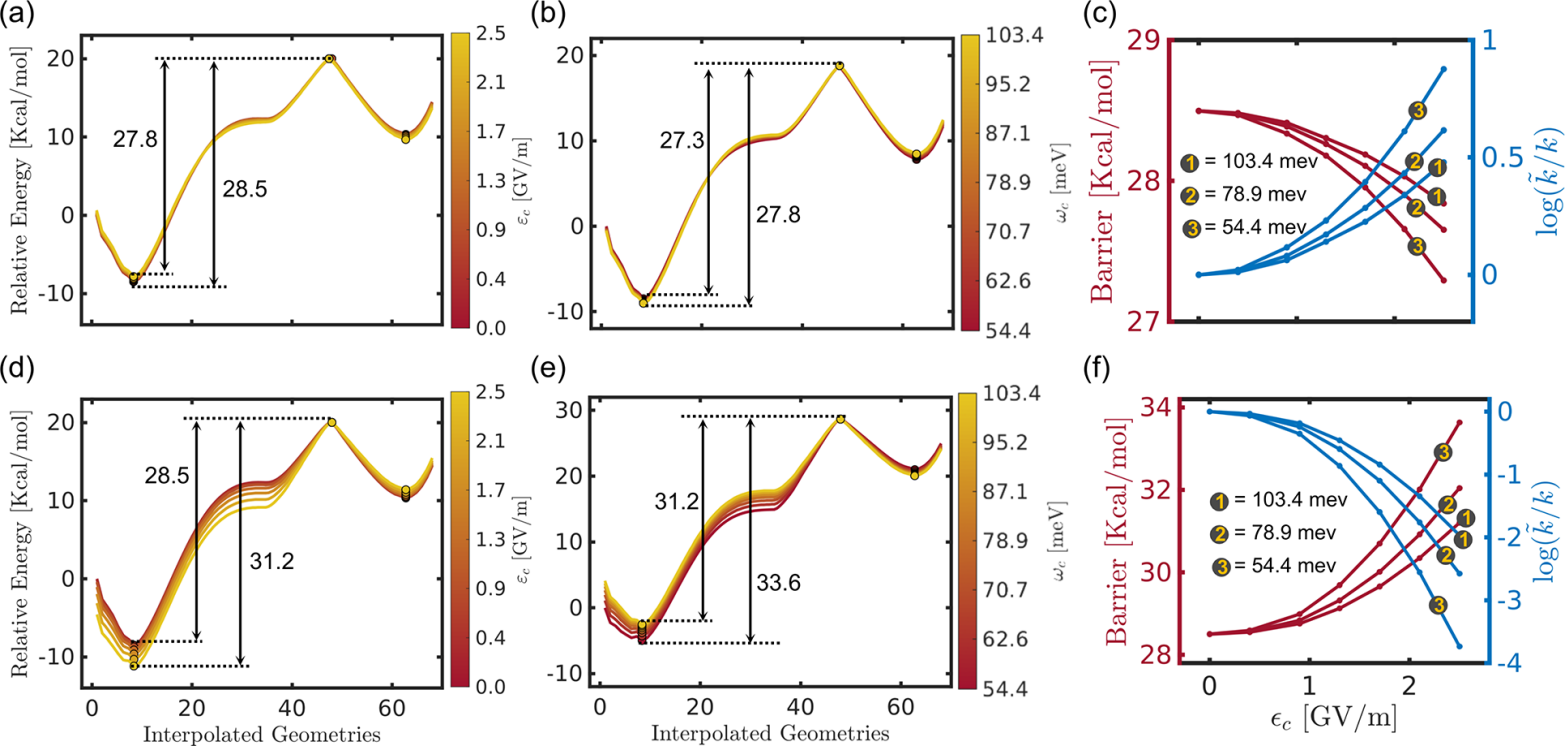
(a) Modified S_1_ energy path for the minimum **q̂**_c_ for different cavity field strengths at ω_c_ = 103.4 meV. (b) S_1_ MEP for different cavity frequencies ω_c_ between 54.4 and 103.4 meV at ε_c_ = 2.5 GV/m. (c) S_1_ path energy barrier (red curves) and the corresponding rate ratio (blue curves) along ε_c_ for three different cavity frequencies, 103.4, 78.9, and 54.4 meV. The description for (d), (e), and (f) is similar to (a), (b), and (c), respectively, except that the cavity mode is polarized along the *Z* direction (along the C–C′/O–O′ bond) for the former and the *X* direction (along the C–O bond) for the latter.

The cavity-altered S_1_ energy profiles for different cavity parameters, along with their activation energies and reaction rates, are shown in Figure 5. The top panel (Figure 5a–c) corresponds to the results of cav-Z-pol, and the bottom panel (Figure 5d–f) is for the cav-X-pol. Unlike the ground state path, both (cav-Z-pol and cav-X-pol) field orientations are found to be affecting the reaction barrier of the S_1_ MEP. As the field strength ε_c_ is increased, the barrier height for the cav-Z-pol decreases from 28.5 kcal/mol (bare molecule) to 27.8 kcal/mol (for ε_c_ = 2.5 GV/mol). Upon decreasing the ω_c_ to 54.4 meV, the barrier energy is further reduced to 27.3 kcal/mol. This effective change in the reaction barrier corresponds to an ≈10-fold increase in the rate of formation of the S_1_ product compared to the bare molecule. The opposite trend has been observed in the case of cav-X-pol, i.e., the reaction barrier increases significantly by increasing the cavity field strength. Here, the coupling dramatically stabilizes the S_1_-min without modifying the transition state energy, and this increases the barrier to 31.2 kcal/mol. With the lowest cavity frequency (ω_c_ = 54.4 meV), the barrier increases to 33.6 kcal/mol due to which the reaction rate becomes extremely slow (≈10^4^ times). Furthermore, the same cavity parameters destabilize the dissociative product region of the S_1_ state. Consequently, the formation of S_1_-prod can be expected to be almost impossible when the cavity mode is aligned with the *X*-axis (cav-X-pol).

**Figure 6:**
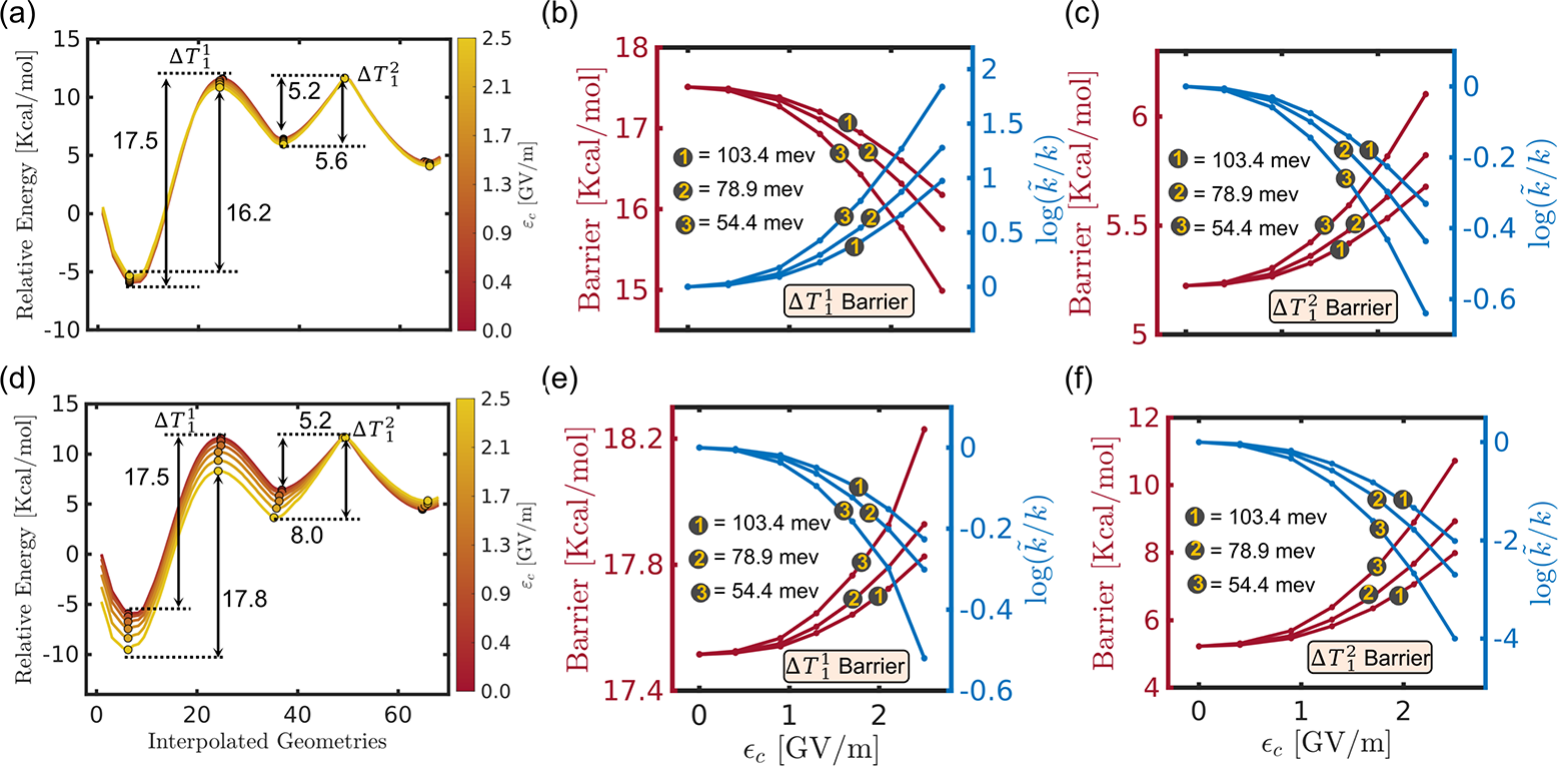
(a) Modified T_1_ energy path for minimum *q̂*_c_ for different cavity field strengths at ω_c_ = 103.4 meV. (b) Δ*T*_1_^1^ barrier (red curves) of the T_1_ path and the corresponding rate ratio (blue curves) along ε_c_ at three different cavity frequencies, 103.4, 78.9, and 54.4 meV. (c) Δ*T*_1_^2^ barrier (red curves) of the T_1_ path and the corresponding rate ratio (blue curves) along ε_c_ for three different cavity frequencies, 103.4, 78.9, and 54.4 meV. The description for parts d, e, and f is similar to parts a, b, and c, respectively, except the cavity mode is polarized along the *Z* direction (along the C–C′/O–O′ bond) for the former and the *X* direction (along the C–O bond) for the latter.

Concerning the triplet channel, there exist two barriers along the pathway, as mentioned above, and these are denoted as Δ*T*_1_^1^ and Δ*T*_1_^2^ in Figure 6. With the cav-Z-pol, the light–matter coupling reduces the first barrier from 17.5 kcal/mol (bare molecule) to 16.2 kcal/mol at ε_c_ = 2.5 GV/m and ω_c_ = 103.4 meV. By changing the cavity frequency to 54.4 meV, the barrier becomes 15.0 kcal/mol (Figure 6a,b). Such a decrease in the activation energy causes the reaction rate to enhance significantly to ≈100 times. Surprisingly, the same cavity environment increases the second barrier from 5.2 to 6.1 kcal/mol at ε_c_ = 2.5 GV/m and ω_c_ = 54.4 meV, which corresponds to the decrease in the reaction rate by ≈5 times (Figure 6c). Note that the difference between the two barrier heights (Δ*T*_1_^1^ – Δ*T*_1_^2^ = 8.9 kcal/mol) for the hybrid cavity–molecule system is smaller than that of the bare molecule (12.3 kcal/mol). As a result, the triplet dissociation rate is expected to depend on both barriers, and the ≈100-fold rate enhancement might be affected due to the increase in Δ*T*_1_^2^. To obtain an accurate estimate of the overall reaction rate, dynamics calculations need to be performed. The results of the triplet path with cav-X-pol are provided in Figure 6d–f. Similar to the S_1_ path, the cav-X-pol interaction lowers the triplet minimum and increases the barrier heights. The greatest effect of the cavity coupling has been observed in the case of Δ*T*_1_^2^ leading the value to 10.7 kcal/mol, while the Δ*T*_1_^1^ becomes 18.2 kcal/mol at ε_c_ = 2.5 GV/m and ω_c_ = 54.4 meV. Correspondingly, the reaction rate drastically reduces by ≈10^4^ times (Figure 6f).

To summarize, the cav-Z-pol orientation favors the formation of excited state (both S_1_ and T_1_) products by reducing the corresponding barriers and stabilizing the dissociative region of PESs (Figures 5a and 6a). The cav-X-pol orientation, on the other hand, raises the barrier heights and inhibits the excited state dissociation process (Figures 5d and 6d). The thermal decomposition of dioxetane forms the excited state formaldehyde predominantly in the triplet electronic state. Despite the efficient, spin-allowed, population transfer to the S_1_ state, only a small fraction of the singlet excited products were observed, primarily because of the involved large barriers.^30,31^ The light emission from the triplet state involving the T_1_ → S_0_ (spin-forbidden) transition is relatively weak, which is one of the main reasons for the observed low chemiluminescence yield in dioxetane. Eventhough both the triplet and singlet paths become more accessible with cav-Z-pol, S_1_ dissociation being the spin-favorable process, an increase in the quantum yield of singlet excitation and consequent intense light emission can be expected.

**Figure 7:**
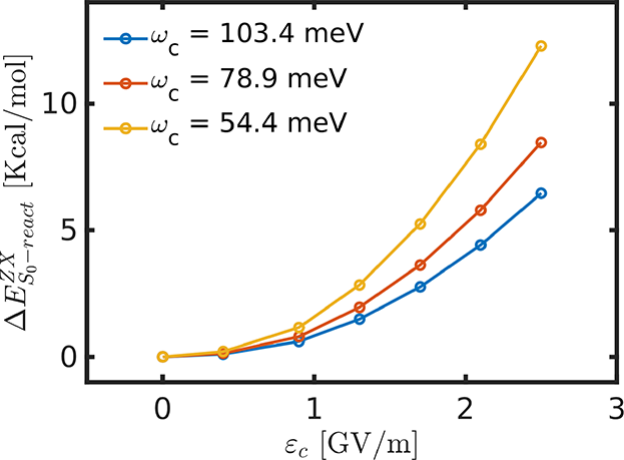
Variation in Δ_S_0–react__^ZX^ (difference in energy of the reactant between cav-Z-pol and cav-X-pol orientations) with respect to the cavity field strength for three different cavity frequencies.

The kinetics of the reaction are influenced by two competing effects: While the cav-Z-pol orientation lowers the barrier slightly, the cav-X-pol orientation raises the barrier significantly. In a randomly oriented sample, the molecules could reorient themselves to cav-Z-pol in order to undergo the reaction with a smaller barrier, and this may lead to an overall faster reaction rate. However, if the energy difference between the reactants for different orientations is taken into account, an overall slowdown of the reaction may be observed: Figure 7 shows Δ*E*_S_0–react__^ZX^ (*E*_S_0–react__^Z^ – *E*_S_0react__^X^), which denotes the energy difference between reactants in cav-Z-pol and cav-X-pol orientation for varying field strength ε_c_. The cav-X-pol orientation is stabilized more than the cav-Z-pol orientation, which could potentially lead to a preferred orientation of molecules. In this scenario, the larger barrier of the cav-X-pol orientation would then lead to an overall slowdown of the reaction. However, additional detailed studies considering both orientations are required to obtain a more accurate picture of the cavity effects on such a randomly oriented scenario.

**Figure 8:**
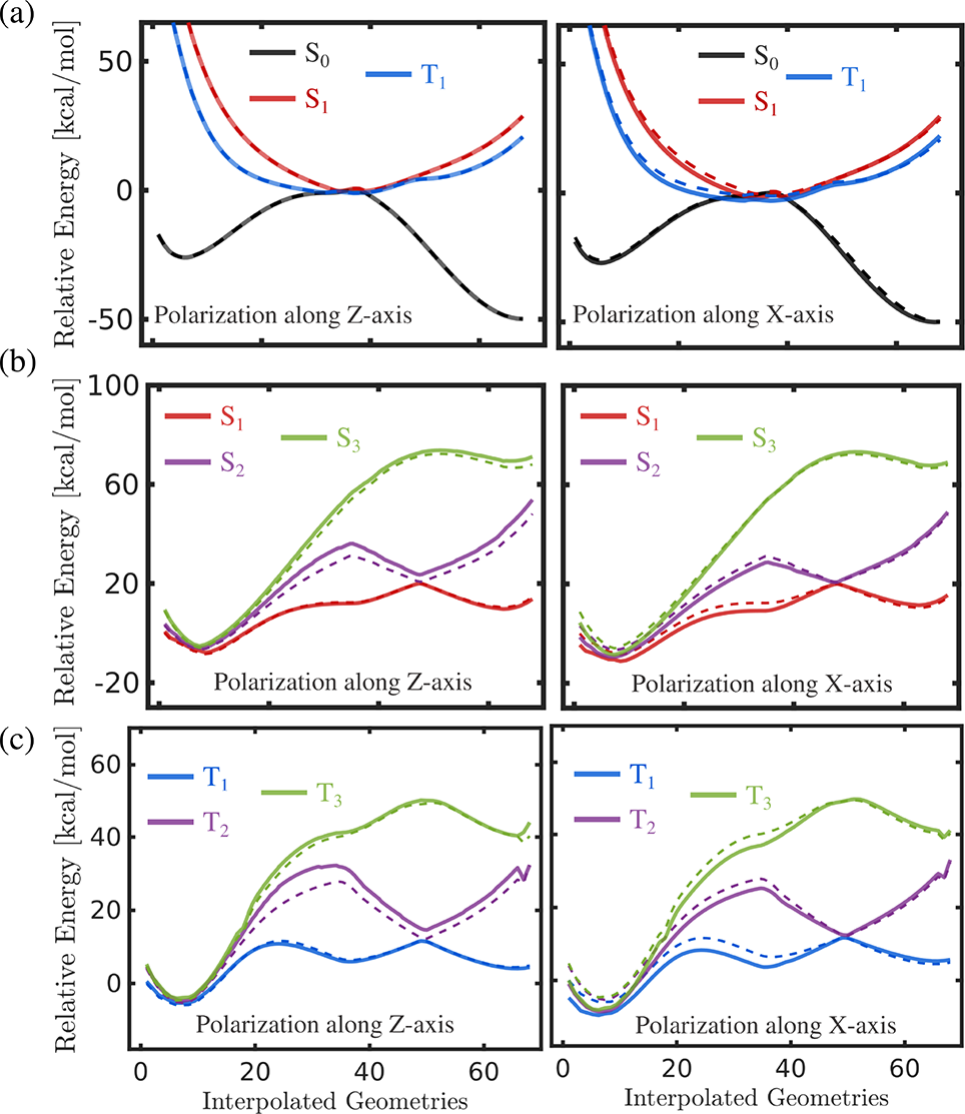
Electronic energies of (a) S_0_, S_1_, and T_1_ states along the S_0_ MEP, (b) S_1_, S_2_, and S_3_ states along the S_1_ MEP, and (c) T_1_, T_2_, and T_3_ states along the T_1_ MEP. Dashed curves are for the bare molecule, and the solid curves represent the paths for the hybrid cavity–molecule system at ω_c_ = 103.4 meV and ε_c_ = 2.5 GV/m.

We now discuss the modified energies of other close-lying electronic states along the computed ground and excited state MEPs. The S_0_ energy profile, with S_0_, S_1_, and T_1_ states, is almost unaffected by the cav-Z-pol orientation (Figure 8a, left panel). This can be anticipated from the corresponding PES (Figure 4a) where the MEP shift along *q̂*_c_ is negligible. The cav-X-pol orientation, however, brings down the S_1_ and T_1_ energy curves. Because of this, the ground and excited states intersect along the S_0_ MEP at a smaller reaction coordinate value as compared to that of the bare molecule (Figure 8a, right panel). This allows the molecule to populate excited states even before reaching the S_0_-TS. Nevertheless, the subsequent dissociation on the S_1_ or T_1_ state is not probable due to the large barriers generated by the cav-X-pol orientation, as described above. In the case of S_1_ MEP, the cav-Z-pol causes the upward shift in the energy curves corresponding to the S_2_ and S_3_ states (Figure 8b, left panel). An impact of this shift is the increase in the energy gap between the S_2_ and S_1_ states. The consequent lifting of S_2_/S_1_ degeneracy may reduce the corresponding nonadiabatic effects and accelerate the formation of S_1_ dissociative product. Similar effects were observed for the triplet channel, where the T_2_/T_1_ degeneracy breaks in the presence of a cavity with a cav-Z-pol orientation (Figure 8c, left panel). The cav-X-pol also alters the energy curves of the excited state paths, but the S_2_/S_1_ and T_2_/T_1_ degeneracies are still preserved (right panels in Figure 8b,c); thereby no further changes can be expected for the dissociation from the S_1_ and T_1_ states.

The role of cavity photon decay and dephasing was not considered in this work. In a recent theoretical study by Rubio and co-workers on cavity influences on thermal Diels–Alder reactions, the impact of cavity losses have been found to be small,^88^ although several other studies have reported the significant influences of photon loss on photochemical reactions.^118−120^ In addition to the photon decay, pure dephasing has also been found to influence the polaritonic effects.^121^ A more detailed study with the inclusion of these energy losses within the cavity and dephasing would be required to ascertain their effects on the dynamics of the chemiluminescent processes.

## Conclusions

In this theoretical study, we explored the polaritonic effects on the chemiluminescent reaction of dioxetane by coupling the molecule with a single cavity mode. Here, the strong coupling effects have been explored for two different cavity environments, where the polarization of the cavity mode is aligned to a particular direction of the molecule. Our results suggest that the optical cavity can either accelerate or completely inhibit the formation of excited state products, depending on the molecular orientation with respect to the field polarization. When the cavity mode is polarized along O–O′ bond or the *Z* direction (cav-Z-pol), the ground state is very similar to that of the bare molecule where S_0_, S_1_, and T_1_ states become degenerate during the thermal decomposition process (Figure 8a, left panel). Consequently, the populated S_1_ and T_1_ states exhibit dissociation barriers of 28.5 and 17.5 kcal/mol, respectively, for the bare molecule, and cav-Z-pol alters these values to 27.3 and 15.0 kcal/mol, respectively, at ω_c_ = 54.4 meV and ε_c_ = 2.5 GV/m. This effective change in the excited state barriers corresponds to the enhancement of the reaction rate by ≈10 times for the S_1_ path and ≈100 times for the T_1_ path (Figures 5c and 6b). It should be noted that the triplet channel has a second barrier (Δ*T*_1_^2^), which increases from 5.2 kcal/mol (bare molecule) to 6.1 kcal/mol (cav-Z-pol) and might affect the overall triplet dissociation rate (Figure 6c). The cav-Z-pol orientation, furthermore, shifts the S_2_ (T_2_) energy curve along the S_1_ (T_1_) MEP, causing the increase in energy gap between the two states, and lifts the S_2_/S_1_ (T_2_/T_1_) degeneracy. This reduces the possible nonadiabatic effects and favors the dissociation process on S_1_ and T_1_ states. Another impact of the rate enhancement, particularly for the S_1_ channel, is the generation of more singlet excited products that efficiently emit light and increase the chemiluminescence quantum yield.

Contrary to the cav-Z-pol, the cavity mode polarization along the C–O bond (cav-X-pol) significantly raises the barrier heights for both the T_1_ and S_1_ MEPs. The corresponding activation energies at ω_c_ = 54.4 and ε_c_ = 2.5 GV/m are 18.2 kcal/mol (Δ*T*_1_^1^) and 10.7 kcal/mol (Δ*T*_1_^2^) for the triplet path and 33.6 kcal/mol for the singlet channel. With these energy barriers, the dissociation rate, for both T_1_ and S_1_ states, roughly decreases by 4 orders of magnitude as compared to the bare molecule. The energy barrier for the ground state path has also been found to be increased with the cav-X-pol.

Future extension of this work will focus on investigating the chemiluminescent process under different cavity environments that are not considered in the present study, e.g., collective coupling effects, resonance effects between the cavity mode and molecular vibrational transitions, and interaction with multiple cavity modes. Furthermore, cavity photon losses and dephasing can also play prominent roles in modifying the reaction dynamics. Along with these cavity effects, dynamics simulation studies on multidimensional PESs of the hybrid molecule–cavity system are essential for gaining additional details, such as branching ratios, lifetimes, and accurate reaction rates.

## Supporting Information Available

Geometrical comparison of optimized critical points obtained in the present work with the previously reported structures, active orbitals included in the SS- and MS-CASPT2 calculations, excited state energies along S_0_ path, reaction barrier and rates corresponding to the S_0_ path along ε_c_, and Cartesian coordinates for the optimized geometries (PDF)
