# Supplementary material for: Cavity-Modified Chemiluminescent Reaction of Dioxetane
Source: J Phys Chem A. 2023 Oct 17;127(45):9483–94. doi: 10.1021/acs.jpca.3c05664 (PMC10658626; doi:10.1021/acs.jpca.3c05664)
Supplement: Supplementary file 1 — jp3c05664_si_001.pdf [file jp3c05664_si_001.pdf]

Supporting Information

Cavity-modified Chemiluminescent Reaction of Dioxetane

Mahesh Gudem* and Markus Kowalewski*

Department of Physics, Stockholm University, Albanova University Centre, SE-106 91 Stockholm, Sweden

Email: mahesh.gudem@fysik.su.se; markus.kowalewski@fysik.su.se

Table S1: The important geometrical parameters of optimized structures on dioxetane PESs at SS-CASPT2/TZVPP (Present work) and MS-CASPT2/ANO-RCC-VTZP (*Farahani et al., JCTC, 2013, 9, 5404–5411*) levels of theory

Geometry	SS-CASPT2/TZVPP			MS-CASPT2/ANO-RCC-VTZP		
	C–C′ (Å)	O–O′ (Å)	O–C–C′–O′ (Deg)	C–C′ (Å)	O–O′ (Å)	O–C–C′–O′ (Deg)
S_0-react	1.51	1.5	17	1.49	1.51	19
S_0-TS	1.51	2.30	43	1.49	2.28	44
S_0-prod	2.00	3.00	77	—	—	—
S_1-min	1.55	3.00	75	1.53	2.92	75
T_1-min	1.5	3.00	68	1.51	3.02	77
S_1-TS	2.22	3.36	83	2.21	3.25	76
T_1-TS	2.42	3.59	82	2.07	3.25	83
S_1-prod	2.50	4.07	-178	3.41	3.57	180
T_1-prod	2.80	4.12	180	3.45	3.61	179

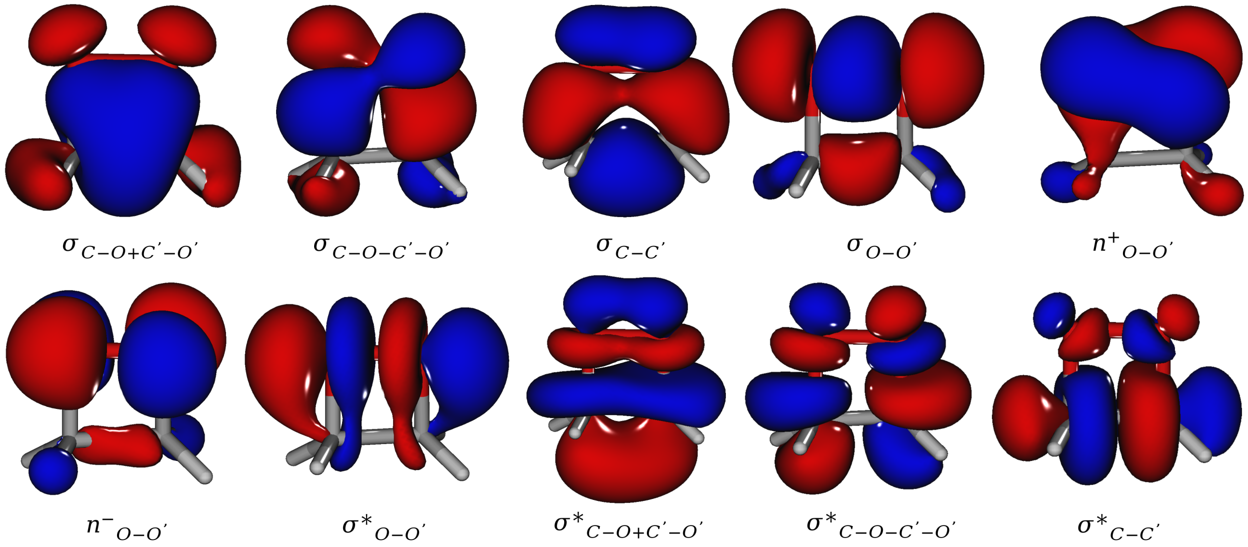

Figure S1: Active orbitals considered for the SS-CASPT2 and MS-CASPT2 calculations. The orbitals (bonding and anti-bonding) corresponding to C–O and C′–O′ bonds are completely mixed. Thus, they are denoted as C–O + C′–O′ and C–O - C′–O′ orbitals.

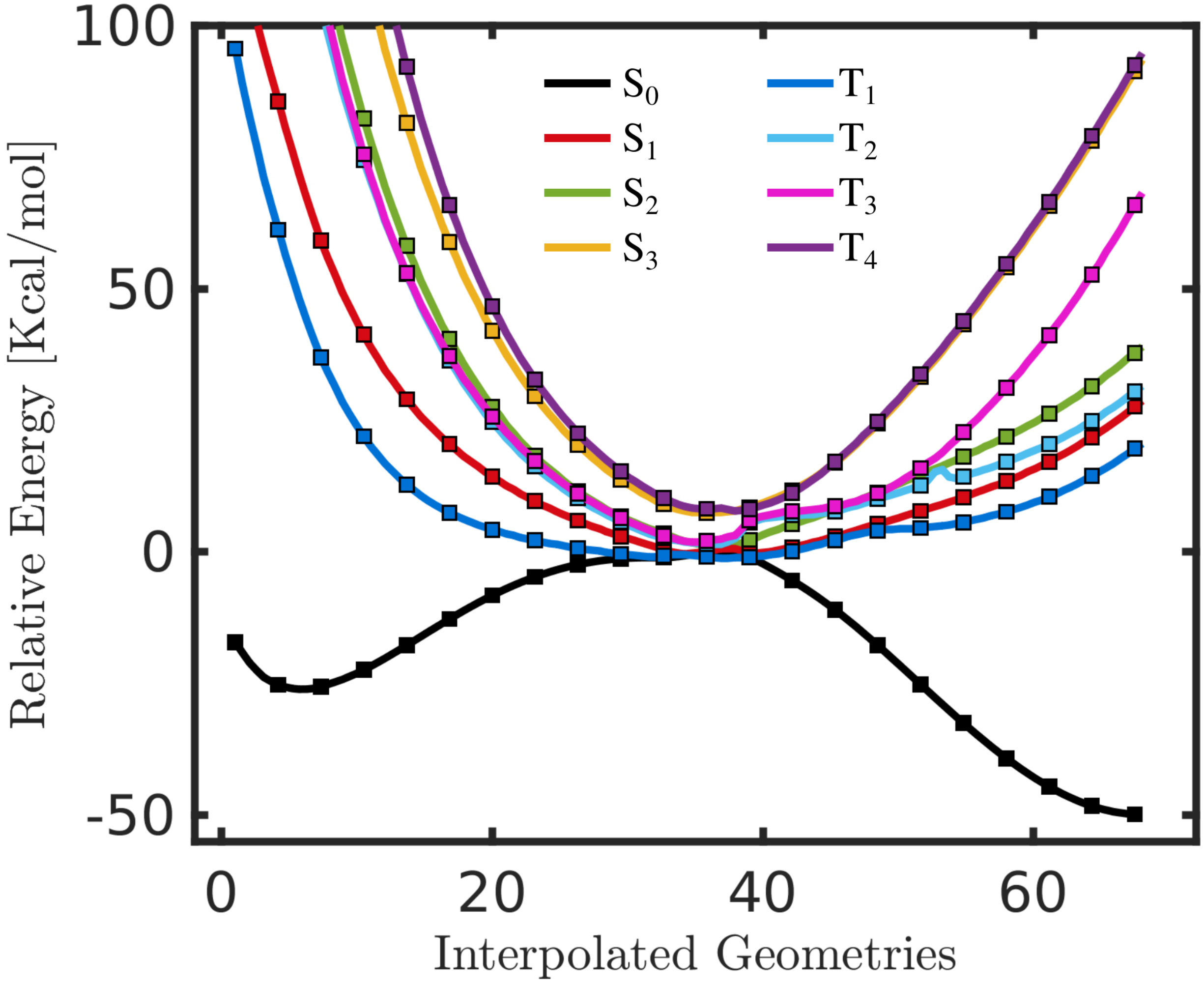

Figure S2: The relative energies for the four lowest singlet and four lowest triplet electronic states along the S_0 path connecting reactant (dioxetane) and dissociative products (formaldehyde), at MS-CASPT2/TZVPP level of theory

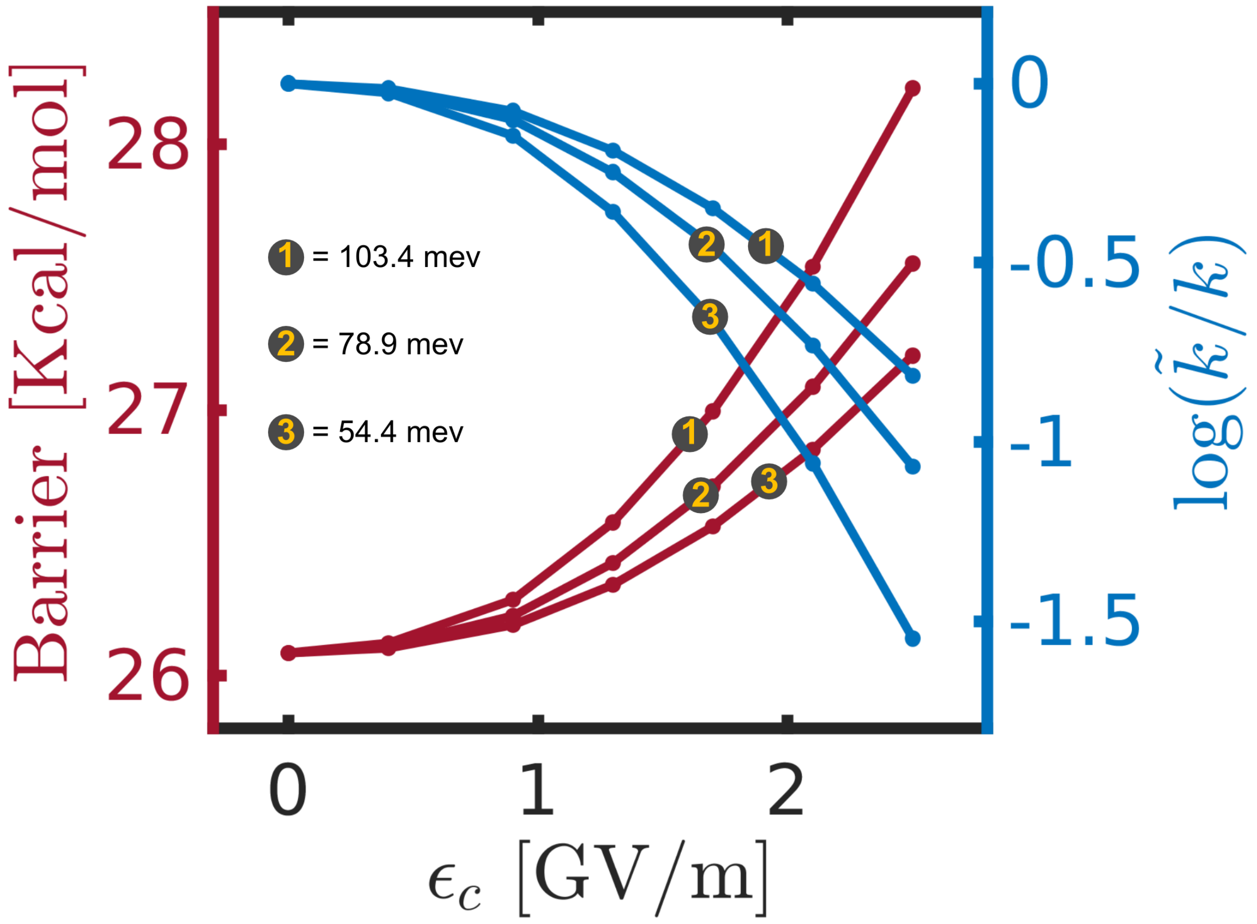

Figure S3: S_0 path energy barrier and the corresponding rate ratio along ε_c for three different cavity frequencies, 103.4 78.9, and 54.4 meV.

Cartesian coordinates for the optimized critical points on S_0, S_1 and T_1 PESs of dioxetane dissociation

S0-react optimized geometry obtained at SS-CASPT2/TZVPP

C	0.0048120212	-0.0221716279	-0.7556205828
C	-0.0048299216	-0.0222339125	0.7556063813
H	0.8115359321	-0.5349168156	-1.2693056658
H	-0.9582022866	-0.2547668995	-1.2042916354
H	-0.8116030823	-0.5349466653	1.2692467902
H	0.9581611402	-0.2549698502	1.2042549334
O	0.2231672137	1.4099224093	-0.7184591594
O	-0.2230400166	1.4098833717	0.7185679285

S0-TS optimized geometry obtained at SS-CASPT2/TZVPP

C	-0.0243630095	0.0444226692	-0.7564692196
C	0.0106865334	0.0399506664	0.7487083226
H	0.6545173513	-0.6776265287	-1.2142874288
H	-1.0319328710	-0.0866656586	-1.1490586824
H	-0.6407142813	-0.6742150190	1.2587352073
H	1.0351106000	-0.0992161376	1.1063907130
O	0.4814835861	1.3254976914	-1.0388805008
O	-0.4847879089	1.3236523170	1.0448605986

S0-prod optimized geometry obtained at SS-CASPT2/TZVPP

C	0.0000000000	0.0000000000	0.0000000000
O	0.0000000000	0.0000000000	1.2097888503
C	0.0000000000	1.9535395649	-0.4285827438
O	1.1286058432	2.3650847680	-0.2435783047
H	-0.9377663635	-0.1040772239	-0.5638368004
H	0.9190090566	-0.1172071353	-0.5852768679
H	-0.3112157566	1.5941054249	-1.4217438375
H	-0.8110995913	2.1281668263	0.2850824209

S1-min optimized geometry obtained at SS-CASPT2/TZVPP

C	-0.0098985616	0.1037721242	-0.7733299996
C	0.0098178966	0.1033304006	0.7732415853
H	0.3765613068	-0.8596312943	-1.1190275541
H	-1.0435434686	0.2158629466	-1.1101480648
H	-0.3775405556	-0.8596599139	1.1191028936
H	1.0435192301	0.2142664057	1.1101902439
O	0.7678382993	1.1186284577	-1.2660315094
O	-0.7667651572	1.1190008634	1.2660243950

S1-TS optimized geometry obtained at SS-CASPT2/TZVPP

C	-0.0996232689	0.1512612031	-1.0359690356
C	0.0874939819	0.0965786694	1.1746793936
H	0.1977985535	-0.8566820803	-1.3177712719
H	-1.1568510874	0.3956577292	-1.0898290802
H	-0.2172687756	-0.9574964428	1.1978262017
H	1.1587304015	0.2813832152	1.0336660005
O	0.7002567142	1.0981429356	-1.5133895882
O	-0.6705395193	0.9869557706	1.5507893801

S1-prod optimized geometry obtained at SS-CASPT2/TZVPP

C	0.0000000000	0.0000000000	0.0000000000
O	0.0000000000	0.0000000000	1.3363758896
C	0.0000000000	2.3957259113	-0.7144909784
O	-0.0345878416	2.4313366368	-1.9259609675
H	-0.9349277935	-0.3171506001	-0.4502518497
H	0.9308058338	-0.3295299825	-0.4499423386
H	-0.9109926991	2.4356112227	-0.1058244710
H	0.9449068000	2.4257453736	-0.1591570242

T1-min optimized geometry obtained at SS-CASPT2/TZVPP

C	0.0016238222	0.1033564097	-0.7608070923
C	-0.0016541534	0.1046910480	0.7608835893
H	0.4288293611	-0.8273867351	-1.1535026182
H	-1.0219761456	0.1528980864	-1.1469789663
H	-0.4294928578	-0.8250711546	1.1552097680
H	1.0219797123	0.1542125405	1.1469682059
O	0.7083663068	1.1450349035	-1.3233016802
O	-0.7076870557	1.1478348917	1.3215507838

T1-TS optimized geometry obtained at SS-CASPT2/TZVPP

C	-0.0915727265	0.1619587468	-0.9802994471
C	0.0879834428	0.0994154466	1.1069317256
H	0.1822161260	-0.8557709579	-1.2583435335
H	-1.1489081061	0.4091514190	-1.0501851048
H	-0.2021933116	-0.9590702820	1.1350648265
H	1.1656522771	0.2876985625	1.0351563910
O	0.6935888984	1.0745331806	-1.4945966586
O	-0.6867696002	0.9778848842	1.5062738008

T1-prod optimized geometry obtained at SS-CASPT2/TZVPP

C	0.0000000000	0.0000000000	0.0000000000
O	0.0000000000	0.0000000000	1.2150183644
C	0.0000000000	2.4394506533	-0.5468825380
O	-0.0001889110	2.8037105412	-1.8103687311
H	-0.9293113003	-0.0518953120	-0.5791738894
H	0.9292685275	-0.0527922348	-0.5792028739
H	-0.9275315634	2.6664944638	-0.0262786401
H	0.9274006720	2.6658978345	-0.0256445947
